# Cervical fibroid – a surgical quandary in gynecology

**DOI:** 10.1093/jscr/rjag161

**Published:** 2026-03-17

**Authors:** Preeti Yadav, Mousumi Das Ghosh, Ruchipriya Samantaray, Archana Barik

**Affiliations:** Department of Obstetrics and Gynecology, Manipal Tata Medical College, Baridih, Jamshedpur 831017, Jharkhand, India; Department of Obstetrics and Gynecology, Tata Main Hospital, Bistupur, Jamshedpur 831001, Jharkhand, India; Department of Obstetrics and Gynecology, Manipal Tata Medical College, Baridih, Jamshedpur 831017, Jharkhand, India; Department of Obstetrics and Gynecology, Tata Main Hospital, Bistupur, Jamshedpur 831001, Jharkhand, India; Department of Obstetrics and Gynecology, Tata Main Hospital, Bistupur, Jamshedpur 831001, Jharkhand, India; Department of Obstetrics and Gynecology, Manipal Tata Medical College, Baridih, Jamshedpur 831017, Jharkhand, India; Department of Obstetrics and Gynecology, Tata Main Hospital, Bistupur, Jamshedpur 831001, Jharkhand, India

**Keywords:** fibroid, myomectomy, MRI, hysterectomy

## Abstract

Cervical fibroids pose unique challenges due to their rarity (0.6% of all uterine fibroids) and location leading to significant anatomical distortion. There will be surgical difficulties due to their proximity to pelvic organs like bladder, ureters, and rectum. This predisposes the surgery for cervical fibroid to complications. Detailed clinical and radiological evaluation of these fibroids help in deciding the treatment approach and mitigate the risk of complication. We present a series of three cases of cervical fibroid with different presentations and management. All these cases were evaluated, and proper pre-operative planning led to complication free surgery. The first and second case presented with huge mass per abdomen and pressure symptoms. Both these cases had pre-operative ureteric stenting followed by definitive surgery. The third case mimicked like a large polyp. This case series will guide surgeons in specific pre-operative planning and surgery in such rare and challenging case.

## Introduction

Uterine fibroids are benign tumors composed of smooth muscle and fibrous connective tissue. They are the most common tumors of uterus among the reproductive age group female with a prevalence of 20%–40% after 35 years of age [1, 2]. The uterine corpus is the common site of fibroids. Cervical fibroids are very uncommon with a frequency of 0.6% of all fibroids [3]. A total of 30% fibroids are asymptomatic, slow growing. When symptomatic, they cause vaginal bleeding, pelvic pain, urinary disorders, pregnancy loss, and in some cases infertility [4]. Huge cervical fibroids presenting as polypoidal mass through vagina may be confused with an incarcerated procidentia or chronic uterine inversion [5, 7]. Cervical fibroids can be anterior, posterior, lateral, and central depending on their position that will further define the presenting symptoms. Ultrasound, magnetic resonance imaging (MRI) and computed tomography (CT) scan play an important role in the management of patients with cervical leiomyomas to detect their number, size, and location. The management of cervical fibroid includes either myomectomy or hysterectomy depending upon patient’s profile. The surgical treatment of cervical leiomyomas poses more difficulty, due to the risk of intraoperative hemorrhage and the potential injuries because of contiguity and dislocation of adjacent organs [6]. Dealing with a case of cervical fibroid is challenging from correct diagnosis to definitive management. Three intriguing cases with various clinical manifestations, diagnostic difficulties, and effective treatment are described here.

## Case 1

**Figure 1:**
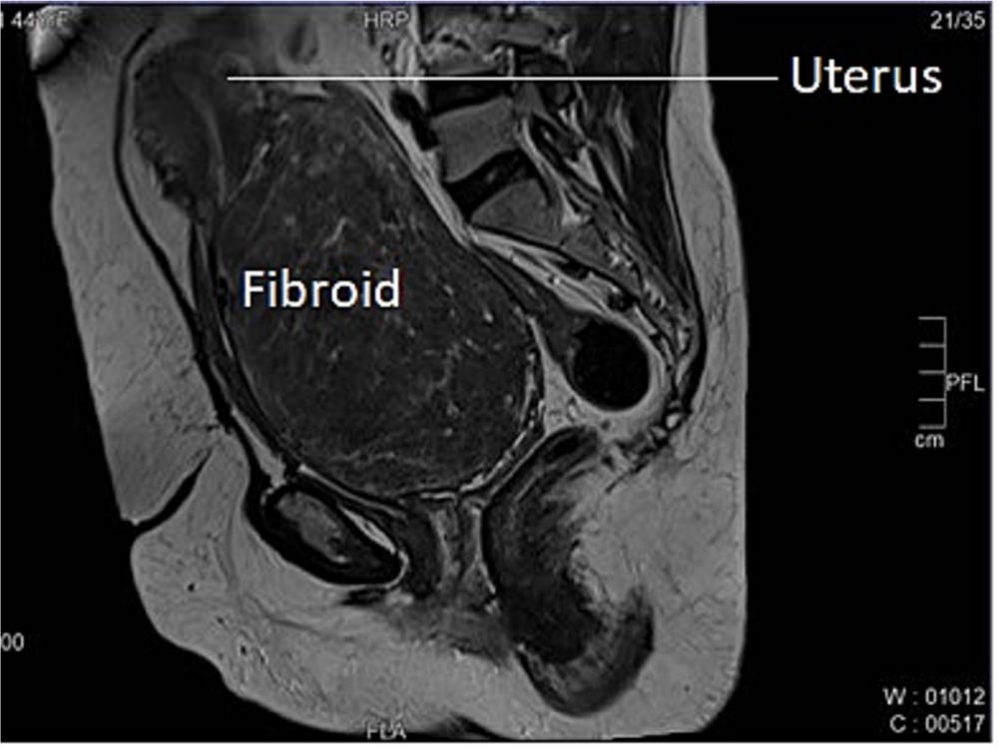
Longitudinal section of MRI image of Case 1 – shows uterus sitting on the top of large cervical fibroid, giving the appearance of a lantern on saint Paul’s dome.

A 46-year-old female, Para 2 Living 2, peri-menopausal, both vaginal deliveries, last childbirth 18 years back, presented to emergency with the incomplete voiding of urine for two days. She was admitted in emergency. She also complaint of pain and abdominal distension for 5–6 months. There was also associated complain of heavy menstrual bleeding for 4–5 months and increased frequency of micturition for 2–3 months. General and systemic examination were normal except for mild pallor. On per abdominal examination, a large globular, firm non-tender mass was felt with well-defined margins and restricted mobility. Size of mass was corresponding to 18–20 weeks gravid uterus. The lower margin of the mass could not be reached. Cervix could not be visualized separately, instead ~8 × 8 cm smooth bulging mass was seen in upper end of vagina. On ultrasonography uterus 10 × 4.7 cm, ET-3 mm, large heterogenous mixed echoic solid mass from cervical portion 16 × 10 cm. Mass was further evaluated with MRI which reported cervical myoma originating from posterior wall ~20 × 9.2 × 17.6 cm displacing the endocervix anteriorly, while the rest of the uterus was displaced antero superiorly. This large well-defined mass occupying whole of cervix and distending the upper vagina, was suggestive of giant cervical leiomyoma, as shown below in Fig. 1. Surgical plan was discussed with the patient and attendants, to which they opted for total abdominal hysterectomy with preservation of bilateral ovaries as her family was completed. After pre-anesthetic workup, the patient was taken up for total abdominal hysterectomy with bilateral ureteric stenting. On opening the abdomen, a giant fibroid of ~20 × 16 cm, was seen occupying the pelvis and abdominal cavity, uterus was sitting on the fibroid, giving the appearance of a Lantern on saint Paul’s dome [6].

**Figure 2:**
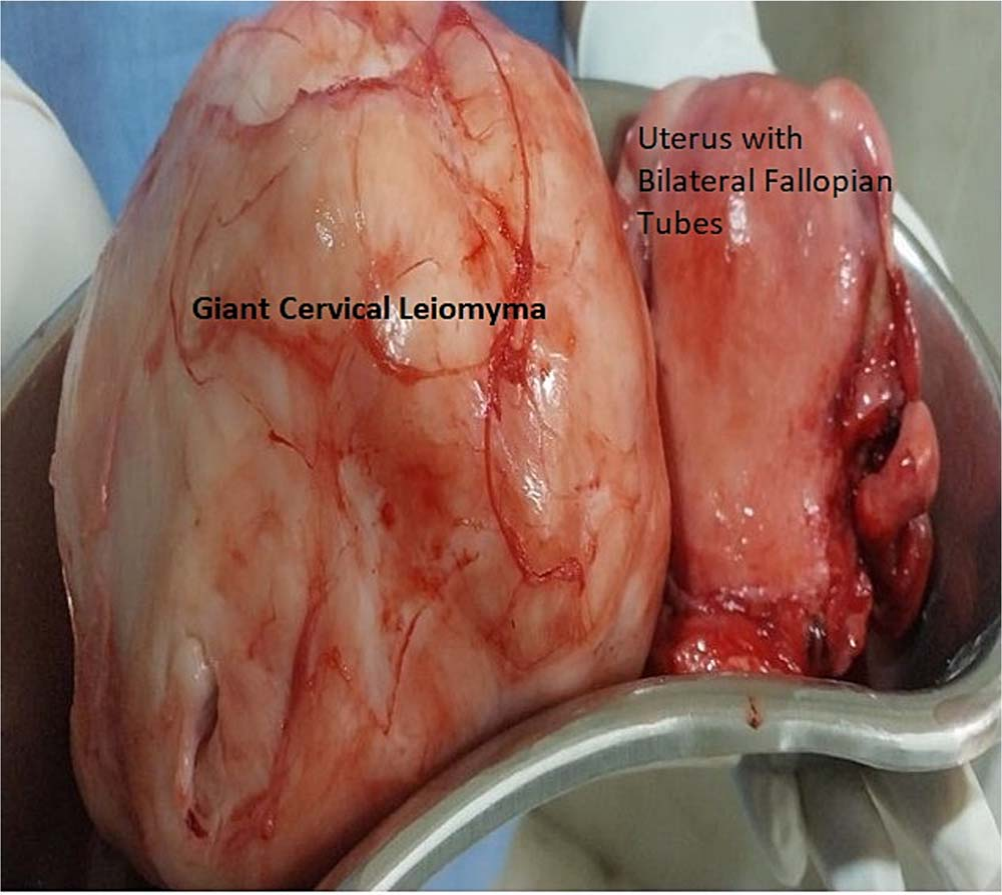
Histopathology specimen image of Case 1 – showing uterus with bilateral fallopian tube removed and large cervical myoma. Here, cervical myomectomy preceded total hysterectomy with bilateral salpingectomy.

Bilateral ureters were dissected, and course identified in the pelvis. Stent in situ was of great assistance in understanding the deviated course of ureter and avoiding any accidental injury to the ureter in clamping and cutting the ligaments and uterine artery. Both anterior and posterior fold of peritoneum identified, separated and then bladder pushed down. Vasopressin was used in a dilution of 0.05 units/ml (20 units in 400 ml of normal saline). Enucleation of fibroid with myoma screw was done after separating the bladder. Bilateral ureters were checked before applying and cutting each clamp. A total abdominal hysterectomy with bilateral salpingectomy was done. Intra-operatively two units of blood were transfused. Figure 2 shows enucleated cervical fibroid with a hysterectomy specimen. Her post-operative period was uneventful. Bilateral ureteric stents were removed on postoperative day-21 after follow-up with urologist. Histopathological examination confirmed cervical leiomyoma with chronic cervicitis.

## Case 2

**Figure 3:**
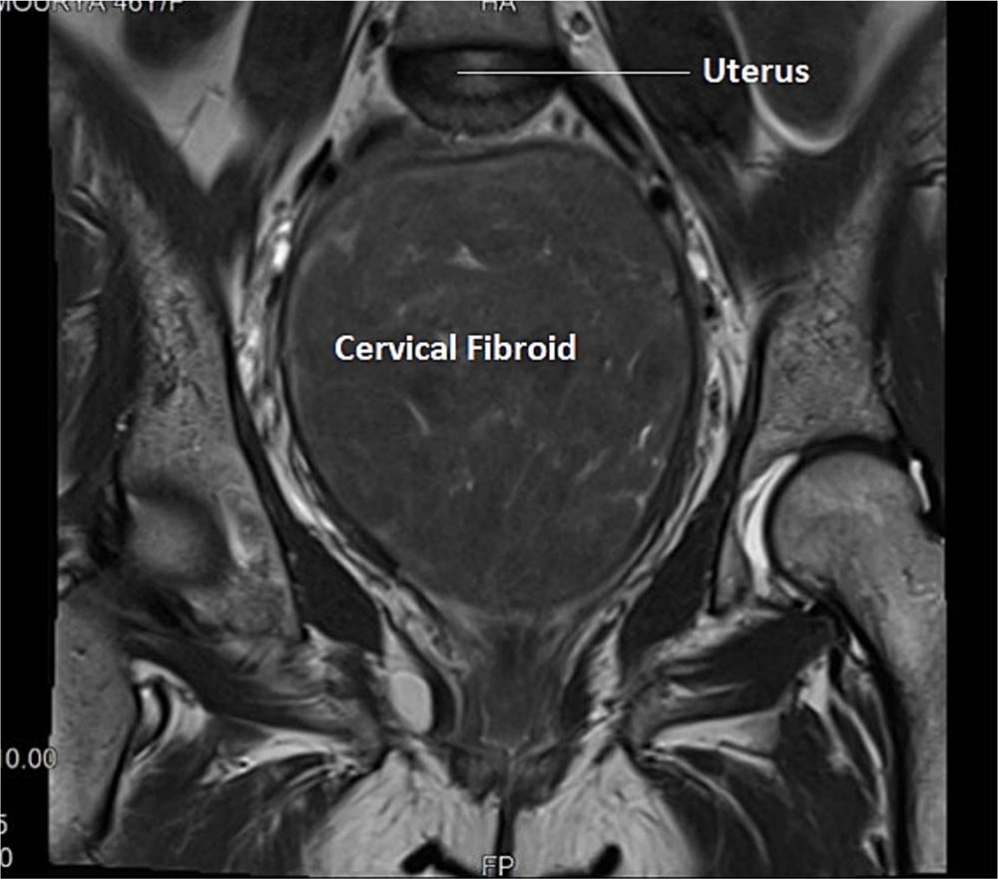
Transverse section of MRI of Case 2 – similar multi-sequential transverse sections aids in clear delineation of course of ureter and its relationship with uterine artery in this case of distorted anatomy because of giant cervical fibroid.

**Figure 4:**
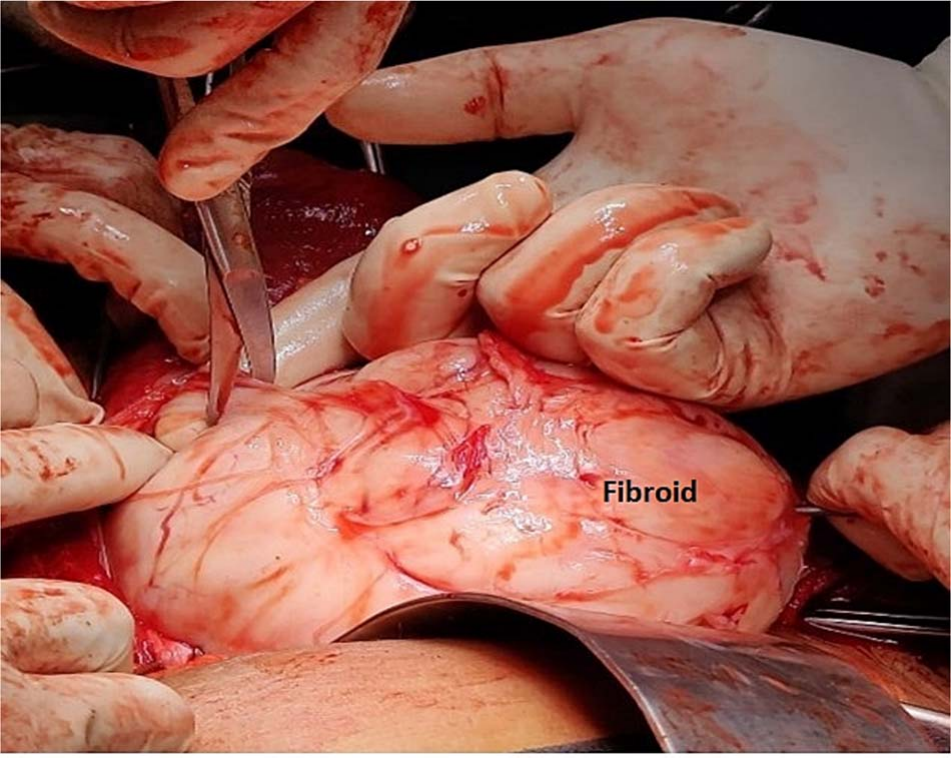
Intra-operative image of Case 2 – myomectomy done with fine dissection with the help of scissors and electrocautery rather than blunt dissections to minimize the blood loss.

A 40-year-old married woman, Para 2 Living 2, with previous two LSCS and last childbirth 9 years back presented to out -patient department with complaints of frequent cycles of 16–20 days interval and heavy menstrual bleeding for 7–8 days every cycle for last 8 months. There is no history intermenstrual spotting. She had laparoscopic cholecystectomy 6 years back. Her last menstrual period was 1 week ago and was associated with heavy vaginal bleeding. General and systemic examination findings were normal. On abdominal examination, a firm mass of 16–18 weeks size was palpated. On local examination, cervix was pulled up, Os could not be felt, a big firm lump was felt in posterior fornix, which moved with uterus, raising the suspicion of cervical fibroid. Ultrasound examination showed an anteverted uterus of 6.8 × 4.1 × 3 cm and ET- 8 mm. Both ovaries were normal. A large cervical fibroid of size 14.2 × 12 × 10 cm was noted. Contrast enhanced MRI of pelvis was done, which showed a uterus of size 7 × 4 × 3 cm, with endometrial thickness of 11 mm, Uterine body displaced superiorly by a large cervical fibroid (15 × 14 × 10 cm), inferiorly protruding into the vaginal cavity as shown in Fig. 3. She was planned for myomectomy after discussing pros and cons of hysterectomy versus myomectomy with couple. Intraoperatively, dense adhesions found between anterior uterine wall, bowel, and omentum. Adhesiolysis was done. Uterus ~20-week size, cervical fibroid of size 15 × 12 cm was present. Bladder densely adhered to lower uterine segment, separated with sharp dissection considering precious cesarean section. Myoma was infiltrated with diluted vasopressin to reduce intra-operative hemorrhage. Myomectomy done with fine dissection with scissors and electrocautery i.e. Fig. 4. Urologist checked the integrity of bladder and ureter at the end of surgery. Patient received one-unit packed cell on postoperative day 1. Patient was discharged on post-operative day. Histopathology confirmed cervical fibroid.

## Case 3

**Figure 5:**
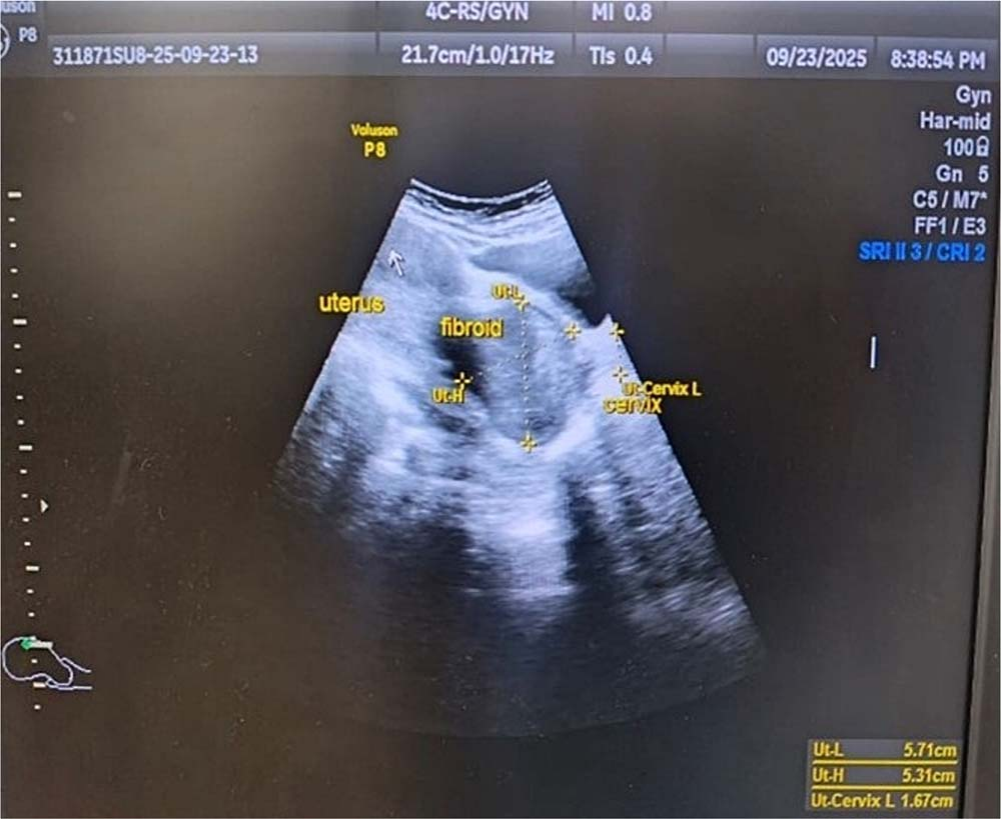
Ultrasonographic image of fibroid polyp of size 5.71 cm × 5.31 cm.

**Figure 6:**
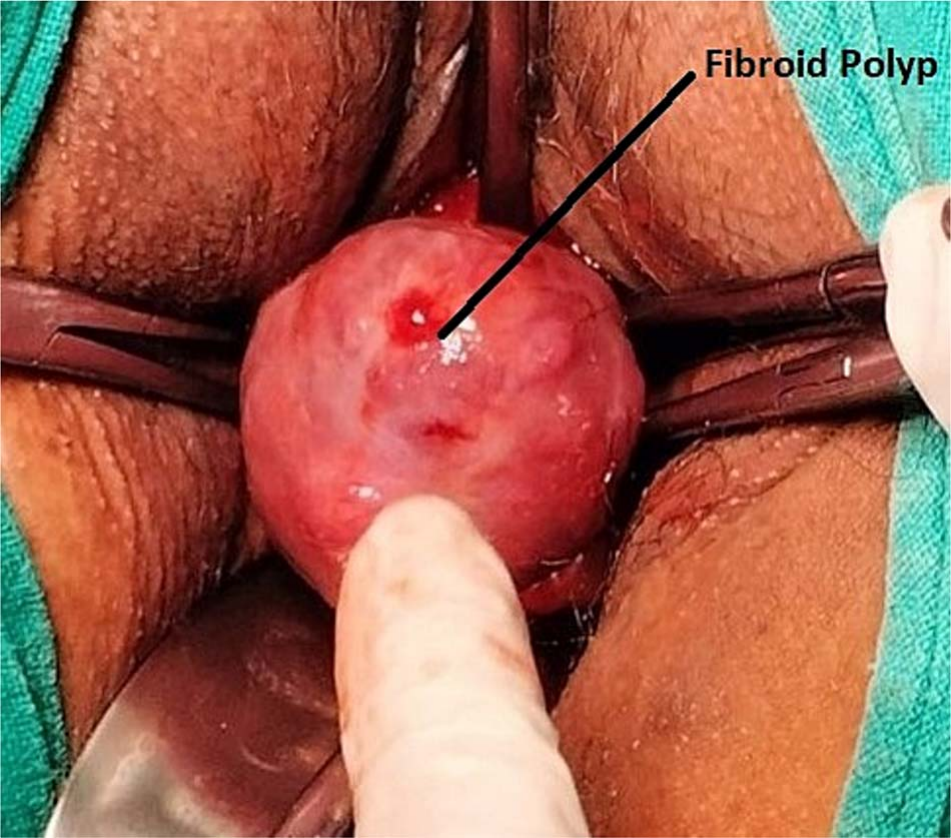
Intra-operative image of Case 3 – cervical fibroid polyp mimicking a malignant mass.

A 43-year-old, married woman, para 3 living 3, presented with a mass coming out from the vagina and gradually increasing in size for 1 year. She also had complaints of offensive vaginal discharge and irregular vaginal bleeding for last 3 months, with passage of clots and intermenstrual spotting. The mass was irreducible in nature, this ruled out differential diagnosis of uterovaginal prolapse. There were no associated bladder or bowel complaints. She had no significant medical or surgical history. She has never used any hormonal therapy. Her last menstrual period was 2 weeks ago. On general examination, she had moderate pallor. A 6 × 6 cm necrotic, irregular, lobulated mass protruding outside the introitus and had variable consistency, varying from soft to firm with some friable areas. These clinical findings raised the suspicion of malignancy. Ultrasound examination showed an anteverted uterus of 8 × 5.5 × 4.3 cm and thin endometrium shown in Fig. 5. Both ovaries were unremarkable. Cervical fibroid of size 5.7 × 5.3 cm was seen on sonography. The patient was transfused with 2 units of packed red cells and worked up for examination under anesthesia and biopsy. Intraoperative findings showed an anteverted uterus of 10 weeks size with a large, grossly necrotic mass of 6 × 6 cm was seen extruding through a healthy-looking cervix by ~2 cm long and 2 cm thick stalk arising from posterior wall of cervix and protruding into the vagina and outside the introitus i.e. Fig. 6. A provisional diagnosis of cervical fibroid polyp was made. Pedicle of fibroid polyp was held with the clamp, cut, and transfixed with vicryl-1 suture. Endometrial curettage and cervical biopsy were done. Intraoperative bleeding was managed with cautery and vaginal packing. She received postoperative analgesics and antibiotics. Post-operative hemoglobin was 9.1 gm/dl. Patient’s postoperative period was uneventful. Patient was discharged on postoperative day 2 with advice to review with histopathology report. The histopathological result of the mass confirmed a benign leiomyomatous polyp of cervical origin. Cervical biopsy and endometrial curettage showed no malignant changes or atypia.

## Discussion

Cervical fibroids are rare benign conditions. It commonly occurs as a single fibroid but can also be multiple. They are either interstitial or subserous. Rarely, can be sub mucosal or polypoidal. In this study, we observed three different types of cervical fibroid, one posterior cervical fibroid, one large central cervical fibroid and one cervical fibroid polyp. As, fibroids arise primarily from smooth muscle thus incidence of large, isolated fibroid in cervical region (Cases 1 and 2) is very uncommon because cervical stroma have very few smooth muscles [6, 7]. Growth of fibroid is estrogen dependent; this explains that in majority of cases fibroids are symptomatic in reproductive age group as was in our all three cases. The presenting symptoms depend upon the location of cervical fibroid. Anteriorly located cervical fibroid pushes the bladder while posterior cervical fibroid flattens the pouch of Douglas and compress rectum against sacrum [7]. Hence, a giant cervical fibroid will have both pressure symptoms i.e. urinary retention and constipation other than abdominal mass and abnormal uterine bleeding. Lateral cervical fibroid that begins at the side of the cervix expands the broad ligament [6]. Cervical fibroids are often diagnosed by CT, MRI or ultrasonography alone, or by combining the two. The diagnosis in our case-1 and case-2 was made by integrating ultrasonography with MRI and case-3 by ultrasonography alone. MRI has been proven superior to ultrasound as it not only provides the complete detail of fibroid by describing its size, location, number, changes but also highlights its relationship with neighboring vital pelvic structures, including the uterine artery, ureters, and bladder [8]. MRI permits better visualization of the lateral & posterior area of pelvis, which are difficult to be evaluated with trans-abdominal or trans-vaginal sonography [7]. In other words, MRI despite being cost-ineffective is very utilitarian as it offers a clear roadmap for surgery. The surgical treatment of cervical leiomyomas is very formidable by virtue of relative inaccessibility and highly vulnerable to complications. Besides, the risk of intraoperative hemorrhage, this surgery carries a significant risk of injury to adjacent vital structures, particularly the ureters, which are frequently displaced and lie close to cervical leiomyomas [3]. At present, there is no universally accepted standard surgical treatment for cervical leiomyomas; consequently, management is individualized based on patient characteristics, reproductive goals, and the expertise of the treating surgeons and centers. In recent years, a limited number of interventional radiology techniques have been reported as alternative options for patients seeking uterine preservation. Cost, lack of expert and available resources are limiting factors for such procedures.

The surgical treatment of symptomatic large cervical myomas includes myomectomy or hysterectomy and is very challenging for the reasons explained earlier. Another concern associated with the surgical treatment of cervical myoma is the intraoperative hemorrhage. Various methods have been developed to reduce the risk of intra-operative bleeding [6]; these procedures include the use of preoperative GnRH agonist, tourniquet method, intraoperative injection of vasopressin into the myometrium, and permanent occlusion of the uterine artery [9]. In our cases we used vasopressin in a dilution of 0.05 units/ml (20 units in 400 ml of normal saline).

Cervical fibroid polyps can present as heavy menstrual bleeding, intermenstrual bleeding, or post-coital bleeding. It can also present as protruding introital mass can with necrosis and foul-smelling discharge. The treatment of choice in majority cases are polypectomy with ensured hemostasis as was done in our case.

## Conclusion

Cervical fibroids are uncommon with varying clinical presentations. Their appropriate diagnosis and definitive management are a challenge. Detailed pre-operative work-up will help in understanding of altered pelvic anatomy. MRI is of utmost significance for clear diagnosis and surgical planning. It aids surgeon in choosing the appropriate surgical techniques and mitigate the potential complications. Hysterectomy, myomectomy or polypectomy (in cases of cervical fibroid polyp), according to the patient’s age and childbearing remains the cornerstone in the treatment of cervical leiomyomas. Since surgery can present difficulties, it should be performed by experienced surgeons.
